# High-throughput cell-based screening reveals a role for ZNF131 as a repressor of ERalpha signaling

**DOI:** 10.1186/1471-2164-9-476

**Published:** 2008-10-11

**Authors:** Xiao Han, Jinhai Guo, Weiwei Deng, Chenying Zhang, Peige Du, Taiping Shi, Dalong Ma

**Affiliations:** 1 Chinese National Human Genome Center, #3-707 North YongChang Road BDA, Beijing 100176, PR China; 2 Laboratory of Medical Immunology, School of Basic Medical Science, Peking University Health Science Center, 38# Xueyuan Road, Beijing, 100191, PR China; 3 Peking University Center for Human Disease Genomics, 38# Xueyuan Road, Beijing, 100191, PR China; 4 Beihua University, 3999 Huashan Road, Jilin City, Jilin Province, 132013, PR China

## Abstract

**Background:**

Estrogen receptor α (ERα) is a transcription factor whose activity is affected by multiple regulatory cofactors. In an effort to identify the human genes involved in the regulation of ERα, we constructed a high-throughput, cell-based, functional screening platform by linking a response element (ERE) with a reporter gene. This allowed the cellular activity of ERα, in cells cotransfected with the candidate gene, to be quantified in the presence or absence of its cognate ligand E2.

**Results:**

From a library of 570 human cDNA clones, we identified zinc finger protein 131 (ZNF131) as a repressor of ERα mediated transactivation. ZNF131 is a typical member of the BTB/POZ family of transcription factors, and shows both ubiquitous expression and a high degree of sequence conservation. The luciferase reporter gene assay revealed that ZNF131 inhibits ligand-dependent transactivation by ERα in a dose-dependent manner. Electrophoretic mobility shift assay clearly demonstrated that the interaction between ZNF131 and ERα interrupts or prevents ERα binding to the estrogen response element (ERE). In addition, ZNF131 was able to suppress the expression of pS2, an ERα target gene.

**Conclusion:**

We suggest that the functional screening platform we constructed can be applied for high-throughput genomic screening candidate ERα-related genes. This in turn may provide new insights into the underlying molecular mechanisms of ERα regulation in mammalian cells.

## Background

Estrogen plays important roles both in reproductive physiology and in numerous human disease states [1,2]. Estrogen exerts its biological actions by binding to two structurally and functionally distinct estrogen receptors (ER), α and β [3-5]. ERα is the major ER in human mammary epithelium. After binding with estrogen, it undergoes an activating conformational change that promotes its homodimerization and nuclear transportation. In the nucleus, the dimer is able to bind, with high affinity, to cis-acting enhancers known as estrogen response elements (ERE), which lie within the regulatory regions of target genes. The classical ERE, originally discovered in the Xenopus vitellogenin gene, consists of a 13-nucleotide inverted palindromic sequence separated by any three nucleotides (5' GGT CAN NNT GAC C 3') [6].

Through its mitogenic effect, estrogen is thought to play a role in breast cancer development [7,8]. As many as 70% of patients with breast cancer are estrogen-receptor positive [9]. ERs may play different pathophysiological roles at different stages of tumor progression, and may also be responsible for the increased incidence of breast cancer following hormone replacement therapy [10]. Although several pathways have been reported, the E2-ER-ERE pathway is believed to be responsible for the mitogenic effect of estradiol and to play a crucial role in regulating its oncogenic expression [11].

ERα activity can be regulated by a multitude of coregulatory factors, termed coactivators and corepressors [12]. Although the biological role of estrogen-mediated activation of genes is well established, the significance of repression has only recently begun to be appreciated. ERα repressors are proposed to provide a counterbalance to estrogen-induced transactivation, and represent a mechanism that may be employed by the cell for tumor suppression [13].

The breast cancer and ovarian susceptibility gene 1 (BRCA1) has been shown to inhibit ERα signaling by repressing the AF-2 domain of ERα, which is linked to the ligand-binding domain. BRCA1 inhibits the estrogen-stimulated expression of pS2, cathepsin D, and a variety of other estrogen-responsive genes in this way [14]. The finding that BRCA1 is a corepressor of ERα provides a potential molecular explanation for the tissue-specific nature of BRCA1-associated cancer. However, cancer-causing mutations of BRCA1 only account for 5–10% of breast cancer. Therefore, it is conceivable that additional factors having a similar corepressor activity may participate in the same regulatory process, and that defects in these may contribute to the etiology of some forms of breast cancer. Thus, our efforts have been devoted to identifying novel human genes, which, like BRCA1, repress ERα activity.

Genetic screen in various model organisms is one means to identify useful genes from the flood of sequence information from the various genome projects [15-17]. Recent advances in cell-based, genetic, high-throughput screening provide an efficient approach to explore gene function and disease relevance [18]. These strategies offer the advantages of speed, cost-effectiveness, genome coverage, and immediate biological relevance [19,20]. In an effort to identify human genes involved in regulating activity of ERα, we constructed a high-throughput, cell-based functional screening platform by linking a response element (ERE) with a reporter gene. This has enabled us to make a quantitative assessment of the effect of candidate genes on the cellular activity of ERα, in the presence or absence of its cognate ligand E2. We describe here the identification and characterization of ZNF131, a novel repressor of ERα mediated transactivation. In addition, the mRNA and protein levels of pS2/TFF1, an estrogen responsive gene, were downregulated by ZNF131.

## Methods

### Plasmids

We constructed a cDNA library from the human RefSeq database . We selected candidate sequences with "Hypothetical", "Predicted", "Putative", or "Unknown" in their definition, or candidates with fewer than three functional research articles. The full-length cDNA of these candidate genes was inserted into the mammalian expression vector pcDNA3.1/myc-His (-) B (pcDB) (Invitrogen, USA) [19]. This cDNA library, containing 570 genes, was then screened. The reporter plasmid pGL4-ERE-LUC was obtained by inserting the following two annealed oligonucleotides between the Xho I-Hind III sites of the multiple cloning site of the luciferase reporter vector pGL4-basic (Promega, Madison, WI): 5' TCG AGA GGT CAC AGT GAC CTA GGT CAC AGT GAC CTA GAT CTG GGC ATA TAA TGGA-3' and 5'-AGC TTC CAT TAT ATA CCC AGA TCT AGG TCA CTG TGA CCT AGG TCA CTG TGA CCTC-3'. This sequence contains two tandem repeats of the consensus ERE oligonucleotide sequence (AGGTCACAGTGACCT) upstream of the minimal human E1B TATA promoter sequence (GGGCATATAAT) [21]. Expression vector hERα was generously provided by Dr. Ann M. Nardulli, University of Illinois, USA. Dr. Richard Baer, Columbia University Medical center, USA, kindly provided the BRCA1 expression plasmid. The pRL-TK plasmid, which contains the Renilla luciferase gene, was used as an internal control and was purchased from Promega (Madison, WI).

### Cell culture, transient transfection and dual-luciferase reporter assay

HeLa cells were maintained in DMEM (HyClone, Logan, UT, USA) supplemented with 10% fetal bovine serum. MCF-7 cells (purchased from ATCC, HTB-22) were maintained in DMEM containing 10% fetal bovine serum and 10 μg/ml insulin (Sigma-Aldrich, St. Louis, MO, USA). All cells were incubated in a 5% CO_2 _incubator at 37°C. Depletion of hormone ligands for steroid receptor activation studies was achieved by cell culture in estrogen-depleted media (prepared without phenol red and supplemented with 10% charcoal/dextran-treated fetal bovine serum [HyClone]).

For reporter gene assays, HeLa cells were seeded on 96-well plates in DMEM lacking phenol red and supplemented with 5% charcoal/dextran-treated FBS. After 18 h, the cells were transfected using the Vigorous transfection reagent (Vigorous Instruments Co., Ltd., China). A total of 95.1 ng plasmid DNA per well were used, comprising 40 ng pGL4-ERE-LUC, 5 ng pRL-TK, 0.1 ng ERα and 50 ng of the candidate gene. 24 h later, the transfected cells were treated with 100 nM 17β-estradiol (E2, Sigma) or 0.1% vehicle (ethanol) for 12 h. Each experiment of non-stimulation and stimulation was performed in triplicate wells. Firefly luciferase activity was detected with the Dual-Glo Luciferase Assay Kit (Promega, USA), using a GENios Pro reader (Tecan, Mannedorf, Zurich, Switzerland), and corrected for transfection efficiency against Renilla luciferase activity.

### Electrophoretic mobility shift assay (EMSA)

The labeled probe was prepared by annealing two oligonucleotides (5'-TCG AAA AGT CAG GTC ACA GTG ACC TGA TCA ATC GA-3' and 5'-TCG ATT GAT CAG GTC ACT GTG ACC TGA CTT TTC GA-3') containing the Xenopus vitellogenin A2 ERE gene, filling in the 3' overhang in the presence of biotin [22,23]. HeLa cells were estrogen-depleted for 3 days, then transfected with ERα and pcDB or the ZNF131 expression construct (pcDB-ZNF131) using a BTX ECM 830 Square Wave electroporator (Genetronics Inc., SanDiego, CA) (120 V, 20 ms, 2 mm gap cuvette). 24 h later, cells were treated with 10 nM or 100 nM E2 for 6 h to increase nuclear localization of the ERα. The nuclear protein extracts were prepared with the NE-PER Nuclear and Cytoplasmic Extraction Reagents (Pierce, Rockford, IL). Protein-DNA binding was measured, briefly, as follows, using the LightShift™ Chemiluminescent EMSA Kit (Pierce, Rockford, IL): equal amounts (10 μg) of nuclear extract protein were used in each reaction, extracts were preincubated with a binding buffer containing 50% glycerol, 50 ng/μl polydI-dC, 1% NP40 and Biotin end-labeled probe at 4°C for 20 min, then at room temperature for 30 min, in a total volume of 20 μl. A 200-fold excess of non-labeled ERE probe was added in some of the experiments. The samples were loaded onto prerun 5% polyacrylamide gels under nondenaturing conditions. After electrophoresis, gel was transferred to Biodyne nylon membrane (Pierce, Rockford, IL, USA). Subsequent steps were performed as instructed by the manufacturer (Pierce).

### Western blot analysis

Cell culture and transfection conditions were identical to those described above for EMSA analysis. After transfection with ERα and the indicated plasmids for 24 h, the cells were incubated with 100 nM E2 or vehicle for 12 h, then lysed with RIPA buffer (150 mM NaCl, 1% NP40, 0.5% sodium deoxycholic acid, 0.1% SDS and 50 mM Tris-HCl with freshly added proteinase inhibitor cocktail) for 30 min on ice. The supernatant protein concentration was measured using a BCA protein assay kit (Pierce). 30 μg total protein was separated by SDS-PAGE (10%). The antibodies used for immunoblotting were purchased from Santa Cruz (CA, USA): goat anti-pS2 (sc-22501), rabbit anti-ERα (H-184), goat anti-cathepsin D (sc-6486), and mouse anti-β-actin (sc-47778). Immunoblotting for β-actin was performed to ensure equivalent amounts of loaded protein. The densitometric analysis of western blots was performed using Bio-Rad Quantity One computer software.

### RT-PCR analysis

RNA was extracted using Trizol reagent (Invitrogen) according to the instructions of the manufacturer. RT-PCR was performed by generating cDNA using the First Strand cDNA Synthesis kit (Invitrogen). Primers used for pS2 were forward 5'-GCT TCT ATC CTA ATA CCA TCG ACG-3' and reverse 5'-ATT TTG AGT AGT CAA AGT CAG AGC-3', which amplified a fragment between positions 243 and 461 of the pS2 mRNA. 36B4 cDNA (coding for the human acidic ribosomal phosphoprotein PO) was used as an estrogen-independent mRNA control [24]. The 36B4 forward primer was 5'-CTC AAC ATC TCC CCC TTC TC-3'; the reverse was 5'-CAA ATC CCA TAT CCT CGT CC-3'. The relative induction levels of pS2 mRNA were calculated using the Automated DNA/RNA electrophoresis HAD-GT12 (eGene, USA).

Expression of the human ZNF131 gene was measured in multiple cell lines and multiple human tissue cDNA libraries. Primers used for ZNF131 were forward 5'-CCG ATT GAA TGA ACA GCG-3' and reverse 5'-CTC CTG TTT GCT CGT GGG-3', while those used for GAPDH were forward 5'-GGG AGC CAA AAG GGT CAT CAT CTC-3' and reverse 5'-CCA TGC CAG TGA GCT TCC CGT TC-3'.

### Statistical analysis

All experiments were repeated three times. All data collected from the luciferase activity assays, EMSA, western Blot analysis and RT-PCR were expressed as mean ± SD. The data presented in some figures are from representative, repeatable experiments. Statistical significance was determined using the Student's two-tailed t-test.

### Bioinformatics analysis of positive genes

Chromosomal localization and gene structure were analyzed with reference to the high-throughput genomic sequence database . Expression profiles were searched for using GNF SymAtlas v1.0.4 . The putative cleavage site of signal peptides was predicted using the Signal P server . PSORT II  software was used to analyze the protein localization. Protein domains were searched by the National Center for Biotechnology Information Conserved Domain Database [19].

## Results

### High-throughput cell-based screening reveals a role for ZNF131 in regulating the activity of ERα

**Figure 1:**
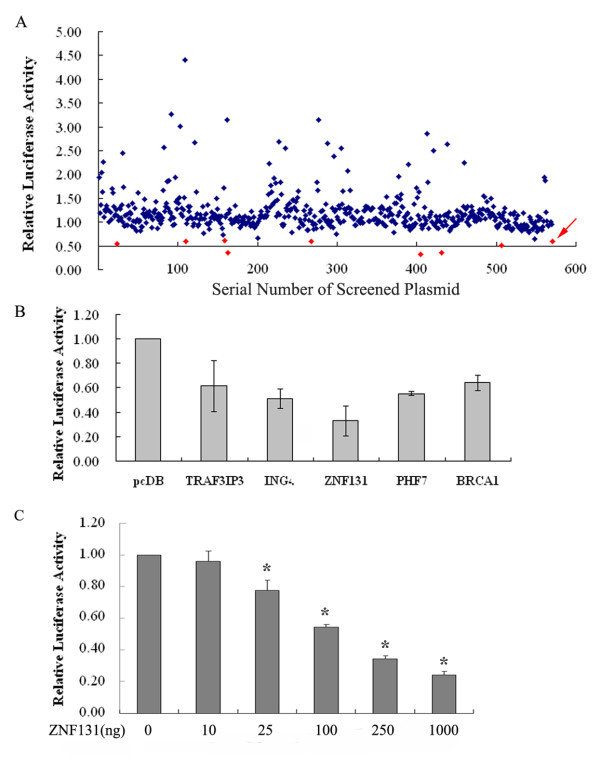
**High-throughput screening of ERE reporter activity inhibitors in HeLa cells**. (A) Candidate genes, cloned into the mammalian expression vector pcDNA3.1 (pcDB), were tested for their ability to inhibit the response, to 17β-estradiol (10 nM), of and ERE-luciferase reporter. Cells cultured in 96-well plates were co-transfected in parallel with plasmids of pGL4-ERE-LUC, pRL-TK, ERα and candidate gene or pcDB. Results were corrected for transfection efficiency against Renilla luciferase activity. Activity in the presence of the empty pcDB vector alone was given an arbitrary value of one. BRCA1, the positive control, yielded a luciferase activity of 0.6 (red arrow). 8 clones in the first round of screening exhibited chemiluminescence values lower than this (red spots). (B) 4 of the 8 candidate genes (TRAF3IP3, ING4, ZNF131, and PHF7) passed a repeat triplicate screening, with ZNF131 identified as the most potent ERα transcription repressor. (C) ZNF131 repressed ERα transcriptional activity in a dose-dependent manner. HeLa cells were transiently transfected with pGL4-ERE-LUC (40 ng), pRL-TK (5 ng), ERα (0.1 ng) and increasing amounts (0, 10, 25, 100, 250, and 1000 ng) of ZNF131 expression plasmids. Cells were treated with 10 nM 17β-estradiol (E2). Data in (B and C) are presented as mean ± SD of three independent experiments. (*, significant difference from control values; P < 0.05)

Our screen for estrogen-suppressing genes involved cotransfecting them into HeLa cells along with both hERα and an ERE-luciferase reporter construct (pGL4-ERE-LUC). Candidate ER-repressor gene cDNAs were cloned into the mammalian expression vector pcDB; this empty vector and BRCA1 were used as the negative and positive control, respectively. Reporter gene activity was first normalized to Renilla luciferase activity obtained by cotransfection of an internal control pRL-TK expression plasmid. As shown in Fig. 1A, in the presence of 17β-estradiol (E2), BRCA1 decreased the transcriptional activity of ERα to an average level of 60%. We decided that candidate genes that were able to repress ERα activity to this degree or further would be selected for further validation. From a library of 570 clones tested, we identified 8 candidate clones in the first round of screening (Fig. 1A, Additional Material). Four of the 8 candidates (TRAF3IP3, ING4, ZNF131, and PHF7) were validated as ERα pathway repressors in the triplicate verification assays (Fig. 1B); their bioinformatics analyses are shown in Table 1. Human zinc finger protein 131 (ZNF131) was identified as the strongest ERα transcription repressor (33%) and was selected for further analysis.

A dose-response assay was constructed by transfecting a range of amounts, from 0 to 1000 ng, of the ZNF131 expression vector in the assay (pcDB was added as necessary to ensure equal amounts of DNA in each transfection). Fig. 1C shows that ZNF131 decreased ERα transcriptional activity in a dose-dependent manner.

### Effect of ZNF131 on the binding of the E2-ER complex to ERE

**Figure 2:**
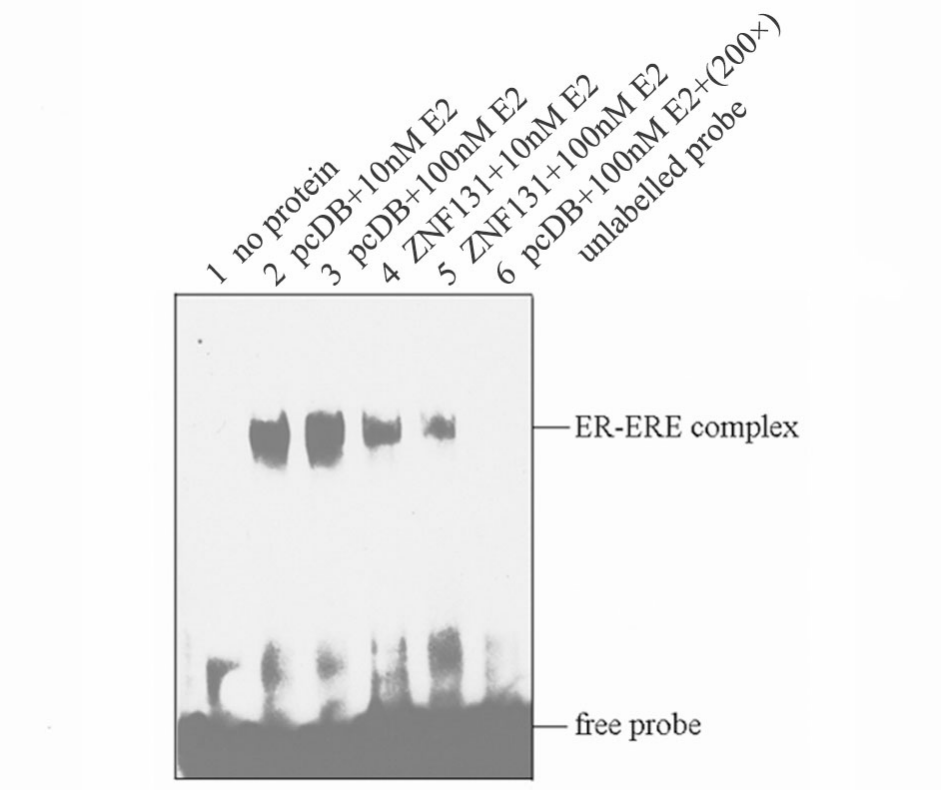
**Electrophoretic mobility shift assay: ZNF131 suppresses ERα-ERE binding activity**. EMSA of ERα-ERE binding activity using nuclear protein from: (lanes 2 and 3) control vector pcDB-transfected cells in the presence of 10 or 100 nM E2; (lanes 4 and 5) ZNF131-transfected cells in the presence of 10 or 100 nM E2; (lane 6) pcDB-transfected cells in the presence of 100 nM E2 where the biotin-labeled ERE probe was competed with 200-fold molar excess of unlabeled ERE probe; and (lane 1) blank control. Results shown are representative of three independent experiments.

To determine how ZNF131 might affect the estrogen signaling pathway, we used an electrophoretic mobility shift assay (EMSA) to evaluate estrogen-dependent binding of ERα to the ERE of the Xenopus vitellogenin A2 gene in nuclear extracts of HeLa cells. As with our screening assay, cells were transfected with exogenous ERα and ZNF131/pcDB plasmids, and then treated with the indicated concentration of E2. Fig. 2 shows that ZNF131 inhibited the binding of the E2-ERα complex to ERE. In nuclear extracts from pcDB-transfected cells, an intense band, corresponding to the ER-ERE complex, was apparent. The intensity of this band was increased in the presence of an increased concentration of E2. Cotransfection with ZNF131 significantly reduced the intensity of the ER-ERE band. A greater reduction in band intensity was seen when the concentration of E2 was raised, suggesting that the inhibition of ERα-ERE binding by ZNF131 is dependent upon the level of E2. The specificity of binding was confirmed by the disappearance of the band in the presence of excess unlabelled oligonucleotide corresponding to ERE.

### ZNF131 inhibits expression of pS2

**Figure 3:**
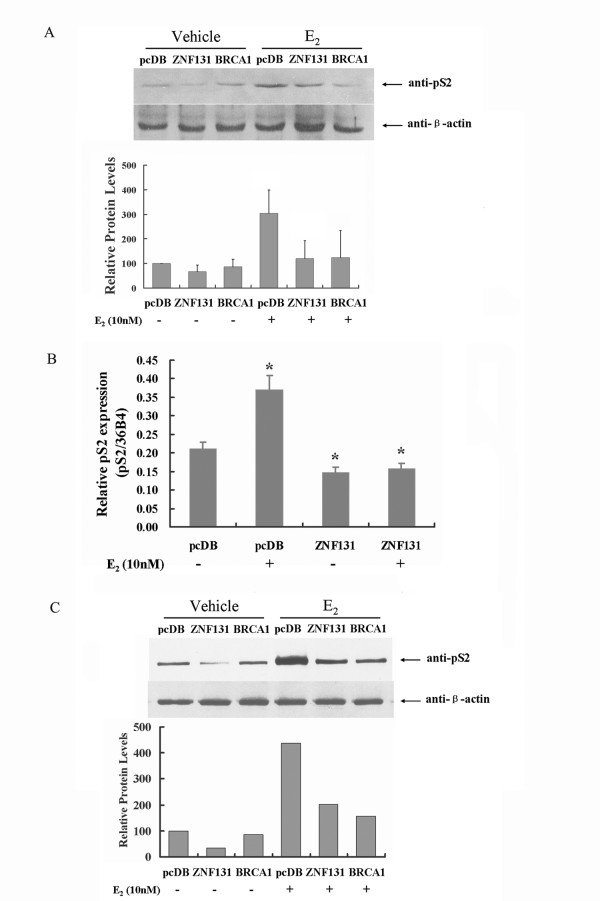
**ZNF131 suppressed pS2 gene expression at both the protein and mRNA levels**. (A) Western blot analysis (top) for pS2 and exogenous ERα expression in HeLa cells transfected with the empty vector (pcDB), ZNF131, or BRCA1, and treated with either E2 (10 nM) or the ethanol vehicle for 12 h. Relative band intensity is shown below. (B) Relative induction of pS2 mRNA in HeLa cells transfected with either pcDB or ZNF131, in the presence (+) or absence (-) of E2. Values are expressed relative to control cultures and represent the mean ± SD of three experiments (*, different from control values; P < 0.05). (C) Western blot analysis of pS2 expression in MCF-7 cells transfected with pcDB, ZNF131 or BRCA1, and treated with either E2 (10 nM) or the ethanol vehicle for 12 h. Relative band intensity analysis using Bio-Rad Quantity One software is shown below. Relative band density values varied, but were qualitatively similar across three independent experiments.

We next determined whether the ZNF131-mediated inhibition of transcriptional activity might extend to other natural estrogen regulatory sites. We first examined the effect of ZNF131 on the expression of pS2, a faithful endogenous ERα-responsive gene whose expression has been widely used as a marker to monitor the regulatory effect of estrogen [25-27]. Fig. 3A shows that the protein level of pS2 was upregulated by the addition of 10 nM estradiol to HeLa cells transfected with pcDB and exposed to exogenous ERα. Coincident overexpression of either ZNF131 or BRCA1 reduced the level of pS2 protein to almost undetectable levels compared to cells transfected with the control vector. RT-PCR and capillary electrophoresis were also utilized to make quantitative analysis. Consistent with the findings from the western blot assay, ZNF131 reduced pS2 mRNA level by 58% in HeLa cells in the presence of 10 nM estradiol (Fig. 3B).

To confirm these observations under biological relevant conditions, we repeated these experiments in MCF-7 cells, in which the expression of pS2 is known to be normally controlled by estrogen (Fig. 3C). We found that, in these cells, pS2 expression in the absence of estrogen was decreased by ZNF131, but not by BRCA1. The addition of estrogen led to an increase in pS2 protein level, which was suppressed in the presence of either ZNF131 or BRCA1 (Fig. 3C).

### Expression profile of ZNF131

**Figure 4:**
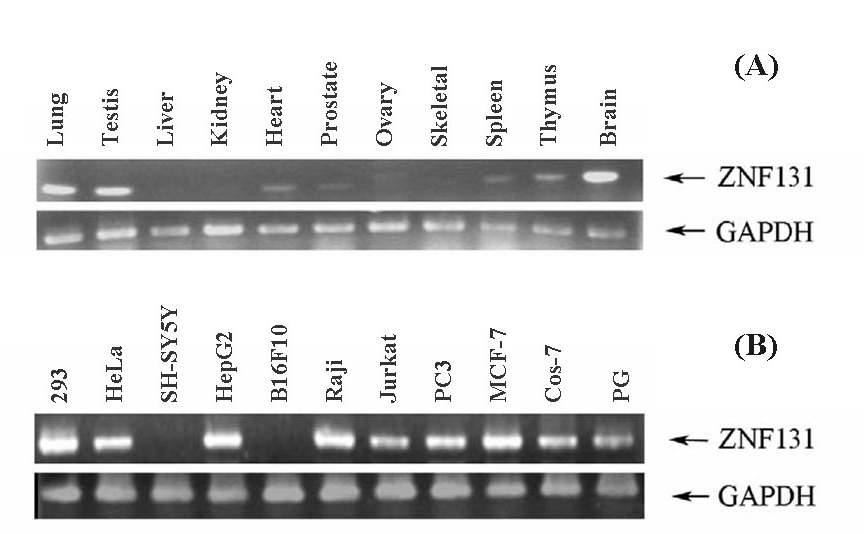
**Expression analysis of ZNF131 by RT-PCR**. (A) Expression profile of ZNF131 in multiple human tissues: lung, testis, liver, kidney, heart, prostate, ovary, skeletal muscle, spleen, thymus, and brain. (B) Expression profile in multiple cell lines, most of which are malignant: 293, HeLa, SH-SY5Y, HepG2, B16F10, Raji, Jurkat, PC3, MCF-7, Cos-7, and PG.

A bioinformatics analysis suggested that ZNF131 is ubiquitously expressed as a member of the POZ-ZF family of transcription factors. Hybridization experiments based on human-tissue-expression blots have shown ZNF131 to be expressed at the highest levels in brain, testis, thymus, and central nervous system [28]. We further studied the distribution of human ZNF131 mRNA across multiple tissues by RT-PCR (Fig. 4A). Consistent with the published results, ZNF131 was detected in many tissues, with highest levels in brain, testis, and thymus. We also found ZNF131 to be highly expressed in lung tissue. Examination of ZNF131 expression in multiple cell lines, showed ZNF131 to be expressed in all but SH-SY5Y and B16F10 cells (Fig. 4B).

## Discussion

Estrogen receptor α (ERα) signaling pathway is important for normal mammary gland development and the onset of breast cancer. The best-characterized corepressor, NcoR, can interact with ERα in the presence of ERα antagonists. Antiestrogen drugs include agents such as tamoxifen, toremefene, raloxifene and fulwestrant. The clinical use of antiestrogens is currently limited to compounds that block the interaction of estrogens of all sources with the ERα. Although these compounds are useful, none identified to date is purely antiestrogenic, and their clinical effectiveness varies [29]. Currently, both tamoxifen and raloxifene are most frequently used in breast cancer chemoprevention. However, tamoxifen's antagonist activity was abolished in NcoR -/- mouse embryo fibroblasts (MEFs) [30], and raloxifene did not provide protection against noninvasive carcinoma as observed in the CORE trial [31]. Thus, finding more ERα repressors might be help in designing therapeutic strategies directed toward epigenetic mechanisms in prevention or treatment of breast cancer.

In the present study, we constructed a high-throughput, cell-based functional screening platform by linking a response element (ERE) to a reporter gene, which allowed us to quantitatively analyze the cellular activity of ERα in cells cotransfected with the candidate genes, in the presence or absence of its cognate ligand E2. We first had to choose an appropriate cell line in which to carry out the assays. Cell transfection efficiency, convenience, and cost were each taken into account. The ERα positive breast cancer cell lines MCF7 and T47D are frequently used to study the influence of genes on the function of ERα. However, the poor transfection efficiency achievable using conventional transfection reagent in these cell lines prompted us to seek a substitute. We chose HeLa cells because they offer good transfection efficiency and are strictly ERα AF-2 permissive [32]. Because HeLa cells are ERα-negative, our assay required cotransfection of a plasmid expressing ERα (hERα). The assay described above confirmed that E2 was able to activate ERα in these cells and ERα exogenously introduced into HeLa cells could restore ERα signaling in response to estrogen.

From an initial screen of a library of 570 human cDNA clones, we identified four genes for further validation, and ultimately identified ZNF131 as the strongest repressor of ERα-mediated transactivation.

ZNF131 is a novel gene whose function is not yet well understood. It was isolated and chromosomally mapped in 1995, together with a group of 15 other novel human zinc finger proteins [33], most of which were considered to be putative candidate genes for developmental and malignant disorders. Analysis of the expression profile of ZNF131 revealed it to be ubiquitously expressed in both the human and mouse [28], with a highest expression in adult testis, thymus, and brain. Our results are in agreement with this expression pattern, with the additional finding of significant expression in lung.

ZNF131 protein has been placed in the superfamily of POK (BTB/POZ and krüppel) proteins, which contain a BTB/POZ domain in their N-terminal region as well as 5 typical C2H2 zinc fingers, and an additional C2HC zinc finger structure, in their C-terminus [28]. In POK proteins, including ZNF131, the BTB/POZ domain putatively confers a specific nuclear localization pattern and mediates transcriptional repression [34]. We confirmed the nuclear localization of human ZNF131 in HeLa cells using a ZNF131-GFP expression construct (data not shown).

The potency of ZNF131 in inhibiting ERα-mediated transcriptional activity in cultured cells was reflected by its dose-dependent inhibition of ligand-dependent transactivation of ERα. EMSA assays showed that the addition of estrogen to HeLa nuclear extracts increased the binding of ERα to ERE, but that overexpression of ZNF131 impaired this activation by destabilizing the E2-ER-ERE complex. The action of ZNF131 was specific, dependent on estradiol, and effective even at high estradiol concentrations.

The MCF-7 cell line was among the first ER-positive human breast cancer cell lines to be characterized as responsive to the mitogenic effects of estrogens in cell culture, as well as in athymic nude mice bearing MCF-7 cell xenografts [35-37]. Further, MCF-7 cells demonstrate estrogen-stimulated expression of a number of well-characterized estrogen-responsive genes, including Cathepsin D and pS2 [14]. Overexpression of ZNF131 in MCF-7 cells led a marked reduction in the expression of pS2 protein, but not of Cathepsin D (data not shown). This suggests that pS2 is a specific target gene of ZNF131. Overexpression of ZNF131 in HeLa cells also repressed pS2 mRNA and protein level.

The pS2 gene product is a well-known estrogen-inducible protein that is expressed in breast and gastrointestinal tissues [38,39]. Expression of pS2 has been used as a marker of estrogen responsiveness in ER containing breast cancer cells, and as a candidate indicator of disease progression to predict the success of antiestrogen therapy. pS2, along with ERα, represent strongly favorable prognostic index in breast cancer [40-42]. Although elevated expression of pS2 predicts a more benign course in breast cancer cases, the mechanism by which this is regulated in human breast cancer cells is not yet well understood. Our finding that overexpression of the ZNF131 protein significantly reduced the level of pS2 in MCF-7 and HeLa cells might contribute to an understanding of the expression of pS2 in normal and neoplastic cells.

## Conclusion

In summary, we utilized a high-throughput screening system and identified a protein, ZNF131, which represses ligand-dependent ERα transactivation and is able to regulate expression of the estrogen-responsive gene pS2. This might provide new insight into designing therapeutic and prognostic strategies of breast cancer. Our observations suggest that ZNF131 as a new ERα repressor correlates with pS2, while cell changes induced by gene overexpression do not necessarily mimic physiological responses in vivo. It is clear that additional studies need to be conducted to understand the regulatory mechanisms and physiological roles of ZNF131. Appropriate in vitro experiments might, first, include the inactivation of ZNF131 through RNAi to decrease endogenous protein level. In addition, ChIP assays might allow further study the mechanism of sequential recruitment of ERα-containing protein complexes to estrogen-target promoters, and allow investigators to generate libraries of repressor-bound promoters.

## Abbreviations

ZNF131: zinc finger protein 131; BRCA1: breast cancer and ovarian susceptibility gene 1; pS2/TFF1: trefoil factor 1; E_2_: estradiol; Erα: estrogen receptor α; pcDB: mammalian expression vector pcDNA3.1/myc-His (-) B; EMSA: electrophoretic mobility shift assay

## Authors' contributions

Han X participated in the design of the study, carried out the high-throughput screen, performed the validation assays, and was pivotal in drafting the manuscript. Guo J participated in constructing the screening system. Deng W participated in the high-throughput screening and carried out the EMSA assay. Zhang C prepared the plasmids used in the screening assay. Ma D, Du P, and Shi T supervised the study and were involved in the conceptualization and writing. All authors read and approved the final manuscript.

## Supplementary Material

Additional file 1 **Table 1.** Bioinformatics analysis of 4 positive genes from the high-throughput screen. Click here for file

Additional file 2 **Supplementary material of the high-throughput screening results.** Click here for file

## References

[B1] Nilsson S, Makela S, Treuter E, Tujague M, Thomsen J, Andersson G, Enmark E, Pettersson K, Warner M, Gustafsson JA (2001). Mechanisms of estrogen action. Physiol Rev.

[B2] Deroo BJ, Korach KS (2006). Estrogen receptors and human disease. J Clin Invest.

[B3] Kuiper GG, Enmark E, Pelto-Huikko M, Nilsson S, Gustafsson JA (1996). Cloning of a novel receptor expressed in rat prostate and ovary. Proc Natl Acad Sci USA.

[B4] Hall JM, McDonnell DP (1999). The estrogen receptor beta-isoform (ERbeta) of the human estrogen receptor modulates ERalpha transcriptional activity and is a key regulator of the cellular response to estrogens and antiestrogens. Endocrinology.

[B5] Ascenzi P, Bocedi A, Marino M (2006). Structure-function relationship of estrogen receptor alpha and beta: impact on human health. Mol Aspects Med.

[B6] Klein-Hitpass L, Kaling M, Ryffel GU (1988). Synergism of closely adjacent estrogen-responsive elements increases their regulatory potential. J Mol Biol.

[B7] Lin CC, Tsai YL, Huang MT, Lu YP, Ho CT, Tseng SF, Teng SC (2006). Inhibition of estradiol-induced mammary proliferation by dibenzoylmethane through the E2-ER-ERE-dependent pathway. Carcinogenesis.

[B8] Chang CJ, Chiu JH, Tseng LM, Chang CH, Chien TM, Wu CW, Lui WY (2006). Modulation of HER2 expression by ferulic acid on human breast cancer MCF7 cells. Eur J Clin Invest.

[B9] Greenwald P (2004). Clinical trials in cancer prevention: current results and perspectives for the future. J Nutr.

[B10] Ito K (2007). Hormone replacement therapy and cancers: the biological roles of estrogen and progestin in tumorigenesis are different between the endometrium and breast. Tohoku J Exp Med.

[B11] Perillo B, Sasso A, Abbondanza C, Palumbo G (2000). 17beta-estradiol inhibits apoptosis in MCF-7 cells, inducing bcl-2 expression via two estrogen-responsive elements present in the coding sequence. Mol Cell Biol.

[B12] Klinge CM, Jernigan SC, Smith SL, Tyulmenkov VV, Kulakosky PC (2001). Estrogen response element sequence impacts the conformation and transcriptional activity of estrogen receptor alpha. Mol Cell Endocrinol.

[B13] Dobrzycka KM, Townson SM, Jiang S, Oesterreich S (2003). Estrogen receptor corepressors – a role in human breast cancer?. Endocr Relat Cancer.

[B14] Zheng L, Annab LA, Afshari CA, Lee WH, Boyer TG (2001). BRCA1 mediates ligand-independent transcriptional repression of the estrogen receptor. Proc Natl Acad Sci USA.

[B15] Moch H, Schraml P, Bubendorf L, Mirlacher M, Kononen J, Gasser T, Mihatsch MJ, Kallioniemi OP, Sauter G (1999). High-throughput tissue microarray analysis to evaluate genes uncovered by cDNA microarray screening in renal cell carcinoma. Am J Pathol.

[B16] Boettner M, Prinz B, Holz C, Stahl U, Lang C (2002). High-throughput screening for expression of heterologous proteins in the yeast Pichia pastoris. J Biotechnol.

[B17] Pritsker M, Ford NR, Jenq HT, Lemischka IR (2006). Genomewide gain-of-function genetic screen identifies functionally active genes in mouse embryonic stem cells. Proc Natl Acad Sci USA.

[B18] Wu CX, Shi TP, Ma DL (2005). Applications of cell-based screening assays in human functional genomics research. Progress in Biochemistry and Biophysics.

[B19] Wang L, Gao X, Gao P, Deng W, Yu P, Ma J, Guo J, Wang X, Cheng H, Zhang C (2006). Cell-based screening and validation of human novel genes associated with cell viability. J Biomol Screen.

[B20] Ma X, Wang X, Gao X, Wang L, Lu Y, Gao P, Deng W, Yu P, Ma J, Guo J, Cheng H, Zhang C, Shi T, Ma D (2007). Identification of five human novel genes associated with cell proliferation by cell-based screening from an expressed cDNA ORF library. Life Sciences.

[B21] Legler J, Brink CE van den, Brouwer A, Murk AJ, Saag PT van der, Vethaak AD, Burg B van der (1999). Development of a stably transfected estrogen receptor-mediated luciferase reporter gene assay in the human T47D breast cancer cell line. Toxicol Sci.

[B22] Chien PY, Ito M, Park Y, Tagami T, Gehm BD, Jameson JL (1999). A fusion protein of the estrogen receptor (ER) and nuclear receptor corepressor (NCoR) strongly inhibits estrogen-dependent responses in breast cancer cells. Mol Endocrinol.

[B23] Rato AG, Pedrero JG, Martinez MA, del Rio B, Lazo PS, Ramos S (1999). Melatonin blocks the activation of estrogen receptor for DNA binding. Faseb J.

[B24] Laborda J (1991). 36B4 cDNA used as an estradiol-independent mRNA control is the cDNA for human acidic ribosomal phosphoprotein PO. Nucleic Acids Res.

[B25] Charafe-Jauffre E, Eisinger F, Mathoulin-Portier MP, Sobol H, Jacquemier J (2001). PS2 expression in BRCA1-associated breast cancers. Anticancer Res.

[B26] Corte MD, Tamargo F, Alvarez A, Rodriguez JC, Vazquez J, Sanchez R, Lamelas ML, Gonzalez LO, Allende MT, Garcia-Muniz JL (2006). Cytosolic levels of TFF1/pS2 in breast cancer: Their relationship with clinical-pathological parameters and their prognostic significance. Breast Cancer Res Treat.

[B27] van Meeuwen JA, Ter Burg W, Piersma AH, Berg M van den, Sanderson JT (2007). Mixture effects of estrogenic compounds on proliferation and pS2 expression of MCF-7 human breast cancer cells. Food Chem Toxicol.

[B28] Trappe R, Buddenberg P, Uedelhoven J, Glaser B, Buck A, Engel W, Burfeind P (2002). The murine BTB/POZ zinc finger gene Znf131: predominant expression in the developing central nervous system, in adult brain, testis, and thymus. Biochem Biophys Res Commun.

[B29] Parker MG (1996). Antiestrogen-estrogen receptor interactions. Cancer Treat Res.

[B30] Jepsen K, Hermanson O, Onami TM, Gleiberman AS, Lunyak V, McEvilly RJ, Kurokawa R, Kumar V, Liu F, Seto E (2000). Combinatorial roles of the nuclear receptor corepressor in transcription and development. Cell.

[B31] Castrellon AB, Gluck S (2008). Chemoprevention of breast cancer. Expert Rev Anticancer Ther.

[B32] Merot Y, Metivier R, Penot G, Manu D, Saligaut C, Gannon F, Pakdel F, Kah O, Flouriot G (2004). The relative contribution exerted by AF-1 and AF-2 transactivation functions in estrogen receptor alpha transcriptional activity depends upon the differentiation stage of the cell. J Biol Chem.

[B33] Tommerup N, Vissing H (1995). Isolation and fine mapping of 16 novel human zinc finger-encoding cDNAs identify putative candidate genes for developmental and malignant disorders. Genomics.

[B34] Donaldson NS, Daniel Y, Kelly KF, Graham M, Daniel JM (2007). Nuclear trafficking of the POZ-ZF protein Znf131. Biochim Biophys Acta.

[B35] Wiese TE, Polin LA, Palomino E, Brooks SC (1997). Induction of the estrogen specific mitogenic response of MCF-7 cells by selected analogues of estradiol-17 beta: a 3D QSAR study. J Med Chem.

[B36] Pawlak KJ, Wiebe JP (2007). Regulation of estrogen receptor (ER) levels in MCF-7 cells by progesterone metabolites. J Steroid Biochem Mol Biol.

[B37] Koibuchi Y, Iino Y, Uchida T, Andoh T, Horii Y, Nagasawa M, Horiguchi J, Maemura M, Takei H, Yokoe T (2000). Regulation of estrogen receptor and epidermal growth factor receptor by tamoxifen under high and low estrogen environments in MCF-7 cells grown in athymic mice. Oncol Rep.

[B38] Rio MC, Bellocq JP, Gairard B, Rasmussen UB, Krust A, Koehl C, Calderoli H, Schiff V, Renaud R, Chambon P (1987). Specific expression of the pS2 gene in subclasses of breast cancers in comparison with expression of the estrogen and progesterone receptors and the oncogene ERBB2. Proc Natl Acad Sci USA.

[B39] Rio MC, Bellocq JP, Daniel JY, Tomasetto C, Lathe R, Chenard MP, Batzenschlager A, Chambon P (1988). Breast cancer-associated pS2 protein: synthesis and secretion by normal stomach mucosa. Science.

[B40] el-Tanani MK, Green CD (1995). Oestrogen-induced genes, pLIV-1 and pS2, respond divergently to other steroid hormones in MCF-7 cells. Mol Cell Endocrinol.

[B41] Thompson AM, Elton RA, Hawkins RA, Chetty U, Steel CM (1998). PS2 mRNA expression adds prognostic information to node status for 6-year survival in breast cancer. Br J Cancer.

[B42] Rody A, Diallo R, Poremba C, Speich R, Wuelfing P, Kissler S, Solbach C, Kiesel L, Jackisch C (2004). Estrogen receptor alpha and beta, progesterone receptor, pS2 and HER-2/neu expression delineate different subgroups in ductal carcinoma in situ of the breast. Oncol Rep.

