# Supplementary material for: High-throughput cell-based screening reveals a role for ZNF131 as a repressor of ERalpha signaling
Source: BMC Genomics. 2008 Oct 11;9:476. doi: 10.1186/1471-2164-9-476 (PMC2577665; doi:10.1186/1471-2164-9-476)
Supplement: Additional file 1 — Table 1. Bioinformatics analysis of 4 positive genes from the high-throughput screen. [file 1471-2164-9-476-S1.doc]

Table 1. Bioinformatics analysis of 4 positive genes from the high-throughput screen.

| Gene name | TRAF3IP3 | ING4 | ZNF131 | PHF7 |
| --- | --- | --- | --- | --- |
| Gene description | TRAF3 interacting protein 3 | inhibitor of growth family, member 4 | zinc finger protein 131 | PHD finger protein 7 |
| Accession No. | NM_025228 | NM_016162 | NM_003432 | NM_016483 |
| UniGene | [Hs.147434](http://www.ncbi.nlm.nih.gov/entrez/query.fcgi?db=unigene&cmd=search&term=Hs.147434) | Hs.108183 | [Hs.559433](http://genome-www5.stanford.edu/cgi-bin/SMD/source/sourceResult?choice=Gene&option=CLUSTER&criteria=Hs.559433) | [Hs.699294](http://www.ncbi.nlm.nih.gov/UniGene/clust.cgi?ORG=Hs&CID=699294) |
| Map | 1q32.3-q41 | 12p13.31 | 5p12-p11 | 3p21.1 |
| Gene ID | [80342](http://www.ncbi.nlm.nih.gov/entrez/query.fcgi?db=gene&cmd=retrieve&dopt=graphics&list_uids=80342) | 51147 | 7690 | 51533 |
| No. of exons | 15 | 8 | 7 | 11 |
| Expression profile | blood,thymus | pituitary gland,tonsil | brain,thymus | testis only |
| (top two) |
| No. of amino acids | 531 | 248 | 589 | 381 |
| pI | 8.66 | 6.95 | 5.02 | 8.58 |
| SignalP | no | no | no | no |
| CCD | tRNA-binding arm,prefoldin | Zinc finger, PHD-type | Zinc finger, C2H2-type | Zinc finger, PHD-type |
| Subcellular location | 21.7 %: cytoplasmic, 21.7 %: nuclear, 17.4 %: vesicles of secretory system | 73.9 %: nuclear | nuclear | 60.9 %: nuclear，21.7 %: mitochondrial，17.4 %: cytoplasmic |
| Functional article | 1 | 11 | 1 | 0 |
